# Molecular and epidemiological characterization of carbapenemase-producing Enterobacteriaceae in Norway, 2007 to 2014

**DOI:** 10.1371/journal.pone.0187832

**Published:** 2017-11-15

**Authors:** Ørjan Samuelsen, Søren Overballe-Petersen, Jørgen Vildershøj Bjørnholt, Sylvain Brisse, Michel Doumith, Neil Woodford, Katie L. Hopkins, Bettina Aasnæs, Bjørg Haldorsen, Arnfinn Sundsfjord, on behalf of The Norwegian Study Group on CPE

**Affiliations:** 1 Norwegian National Advisory Unit on Detection of Antimicrobial Resistance, Department of Microbiology and Infection Control, University Hospital of North Norway, Tromsø, Norway; 2 Microbial Pharmacology and Population Biology Research Group, Department of Pharmacy, UiT The Arctic University of Norway, Tromsø, Norway; 3 Research Group on Host-Microbe Interactions, Department of Medical Biology, UiT The Arctic University of Norway, Tromsø, Norway; 4 Department of Infectious Disease Epidemiology, Norwegian Institute of Public Health, Oslo, Norway; 5 Institut Pasteur, Biodiversity and Epidemiology of Bacterial Pathogens, Paris, France; 6 Antimicrobial Resistance and Healthcare Associated Infections (AMRHAI) Reference Unit, National Infection Service, Public Health England, London, United Kingdom; Ross University School of Veterinary Medicine, SAINT KITTS AND NEVIS

## Abstract

The prevalence of carbapenemase-producing Enterobacteriaceae (CPE) is increasing worldwide. Here we present associated patient data and molecular, epidemiological and phenotypic characteristics of all CPE isolates in Norway from 2007 to 2014 confirmed at the Norwegian National Advisory Unit on Detection of Antimicrobial Resistance. All confirmed CPE isolates were characterized pheno- and genotypically, including by whole genome sequencing (WGS). Patient data were reviewed retrospectively. In total 59 CPE isolates were identified from 53 patients. Urine was the dominant clinical sample source (37%) and only 15% of the isolates were obtained from faecal screening. The majority of cases (62%) were directly associated with travel or hospitalization abroad, but both intra-hospital transmission and one inter-hospital outbreak were observed. The number of CPE cases/year was low (2–14 cases/year), but an increasing trend was observed. *Klebsiella* spp. (*n* = 38) and *E*. *coli* (*n* = 14) were the dominant species and *bla*_KPC_ (*n* = 20), *bla*_NDM_ (*n* = 19), *bla*_OXA-48-like_ (*n* = 12) and *bla*_VIM_ (*n* = 7) were the dominant carbapenemase gene families. The CPE isolates were genetically diverse except for *K*. *pneumoniae* where clonal group 258 associated with *bla*_KPC_ dominated. All isolates were multidrug-resistant and a significant proportion (21%) were resistant to colistin. Interestingly, all *bla*_OXA-48-like_, and a large proportion of *bla*_NDM_-positive *Klebsiella* spp. (89%) and *E*. *coli* (83%) isolates were susceptible *in vitro* to mecillinam. Thus, mecillinam could have a role in the treatment of uncomplicated urinary tract infections caused by OXA-48- or NDM-producing *E*. *coli* or *K*. *pneumoniae*. In conclusion, the impact of CPE in Norway is still limited and mainly associated with travel abroad, reflected in the diversity of clones and carbapenemase genes.

## Introduction

Carbapenemase-producing Enterobacteriaceae (CPE) have emerged as a global public health concern during the last two decades [1, 2]. CPE isolates are usually multidrug-resistant (MDR) or even extensively- or pandrug-resistant (XDR/PDR), resulting in limited antibiotic treatment options [1, 3, 4]. Due to the lack of effective therapy, CPE infections have been associated with high mortality rates [5, 6]. Currently, colistin and various combination regimens are generally used for treatment of CPE infections. However, the clinical evidence is mainly based on case reports and observational retrospective studies [1, 4]. Worryingly, high rates of colistin resistance among CPE have been observed in certain regions [7, 8]. Although colistin resistance is often mutation-based, plasmid-mediated colistin resistance has now also been described [9–14], and observed in CPE isolates [11, 15–17].

The main carbapenemases among Enterobacteriaceae include KPC (Ambler class A), the metallo-β-lactamases NDM, VIM and IMP (Ambler class B), and OXA-48-like enzymes (Ambler class D) [1]. Certain carbapenemases dominate in specific regions and countries, i.e. NDM in the Indian subcontinent, KPC in Italy, Portugal, Israel, Greece and the US, and OXA-48-like in many Mediterranean (e.g. Turkey and Malta) and North African countries as well as some other European countries (e.g. Belgium, France, Germany and Spain) [7, 18–20]. Specific clones or clonal groups (CG) are often associated with specific carbapenemases, while other carbapenemases show a more broad diversity with respect to host genetic backgrounds [2, 21]. The global spread of KPC has mainly been associated with *Klebsiella pneumoniae* sequence type (ST) 258 or CG 258 [2, 21, 22]. In contrast, NDM and OXA-48-like enzymes are broadly distributed in various genetic backgrounds of *K*. *pneumoniae* and *Escherichia coli* and for *bla*_NDM_ there is no clear link to a specific plasmid backbone [2, 21]. For *bla*_OXA-48-like_ there is molecular evidence supporting an association with a specific internationally epidemic IncL plasmid backbone [23–25].

The emergence of CPE in the Nordic countries has mainly been associated with single sporadic cases associated with import [26–36], and the prevalence is low compared with other European countries [7, 19]. However, there are indications of local dissemination unrelated to travel in Denmark [37, 38].

The aim of this study was to analyse the epidemiological, phenotypic and molecular characteristics of CPE isolated in Norway from 2007 to 2014 to understand the molecular epidemiology associated with the emergence of CPE in Norway.

## Materials and methods

### Bacterial strains and demographic data

The study collection consisted of 59 CPE isolates genetically-verified at the Norwegian National Advisory Unit on Detection of Antimicrobial Resistance from 2007–2014. The criteria for submitting isolates to the Unit included reduced susceptibility to carbapenems according to the Norwegian Working Group for Antibiotics (AFA, https://unn.no/fag-og-forskning/arbeidsgruppen-for-antibiotikasporsmal-og-metoder-for-resistensbestemmelse-afa)/Nordic Committee on Antimicrobial Susceptibility Testing (NordicAST) guidelines (www.nordicast.org). In 2012 mandatory reporting of confirmed CPE cases to the Norwegian Surveillance System for Communicable Diseases (MSIS) was established. After confirmation at the Advisory Unit, MSIS and the primary lab are notified. The primary laboratory subsequently notifies the responsible clinician, who also reports data to MSIS. Clinical data were collected from the laboratory requisition. Multiple isolates from the same patient were included in the analysis if they were (i) of different species, (ii) the same species, but harboured a different carbapenemase gene or (iii) if the isolates were of the same species and harboured the same carbapenemase gene, but were identified >1 year apart.

### Phenotypic analysis

Species identification was performed using MALDI-TOF MS (Bruker Daltonik GmbH, Bremen, Germany). MIC profiling was performed using gradient strips (Liofilchem, Roseto degli Abruzzi, Italy/bioMérieux, Marcy-l’Étolie, France) and broth microdilution for colistin using in-house designed premade Sensititre microtiter plates (TREK Diagnostic Systems/Thermo Fisher Scientific, East Grinstead, UK). Interpretation was according to EUCAST clinical breakpoints version 6.0 (www.eucast.org). Non-susceptibility included both the intermediate and resistant categories. The AmpC Confirm kit (ROSCO Diagnostica, Taastrup, Denmark), ESBL combination discs (Becton-Dickinson, Franklin Lakes, NJ, USA), KPC, MBL and OXA-48 Confirm kit (ROSCO Diagnostica) and the in-house version of Carba NP test were used for phenotypic typing of β-lactamases [39, 40].

### Molecular analysis

The presence of carbapenemase genes was initially determined by various PCRs for *bla*_KPC_, *bla*_IMI_, *bla*_VIM_, *bla*_NDM_, *bla*_IMP_, *bla*_GIM_, *bla*_SPM_, *bla*_SIM_ and *bla*_OXA-48-like_ [41–44]. WGS was performed on all isolates using the MiSeq platform (Illumina, San Diego, CA, USA) according to the manufacturer’s instructions. Briefly, genomic DNA was purified using the GenElute bacterial genomic DNA kit (Sigma-Aldrich, St. Louis, MO, USA). DNA libraries were prepared using Nextera/Nextera XT kits (Illumina) followed by paired-end sequencing. Contigs were assembled using SPAdes [45] through the iMetAMOS extension [46] of the MetAMOS package [47]. The presence of resistance genes/mutations, carbapenemase genes and single nucleotide polymorphisms (SNP) variations were determined using a customised algorithm that uses Bowtie 2 to map reads against a locally curated reference database and assembled from publically accessible databases. The database comprised sequences for all reported carbapenemase variants. Samtools was used to generate an mpileup file [48] which was then parsed based on read depth (> 10 reads per base) and base-call agreement (> 90%) to determine the base type at each nucleotide position relative to the closest reference sequence. Presence of reported carbapenemase variants were defined based on 100% identity across the whole length of the corresponding reference gene.

STs of *Klebsiella* spp., *E*. *coli* and *Enterobacter cloacae* complex were determined from WGS data using the *Klebsiella* MLST database (http://bigsdb.pasteur.fr/klebsiella/klebsiella.html), EnteroBase (http://enterobase.warwick.ac.uk/species/index/ecoli) for *E*. *coli*, and the *E*. *cloacae* MLST database (http://pubmlst.org/ecloacae). Core genome MLST (cgMLST) was performed on *K*. *pneumoniae* isolates using 694 loci as previously described [22]. A phylogenetic tree was constructed based on the concatenated sequence alignments using RAxML [49] and FigTree (http://tree.bio.ed.ac.uk/software/figtree/).

### Genbank accession numbers

WGS data have been deposited at the National Center for Biotechnology Information (NCBI) under BioProject PRJNA295003.

### Ethical considerations

The study was reviewed and approved by the Regional Committee for Medical and Health Research Ethics North (reference no. 2016/2122/REK Nord and 2017/146/REK Nord) and the Data Protection Officer at the University Hospital of North Norway (reference no. 2017/1562). The need for patient consent was waived by the Regional Committee for Medical and Health Research Ethics North (reference no. 2017/146/REK nord)

## Results

### Bacterial isolates

In total 59 CPE were identified from 53 patients of which 44 were hospitalized patients. Samples from eight patients were taken at general practitioners or in other health care institutions (e.g. elderly care homes). For one patient no information was obtained. Of the 53 patients, four had multiple CPE isolates belonging to different species or different STs. One patient had four *bla*_NDM-1_-positive strains of different species (*Proteus mirabilis*, *Providencia stuartii*, *Citrobacter* sp. and *K*. *pneumoniae*) isolated within a four-month period. Another had *bla*_KPC-2_-positive *K*. *pneumoniae* and *Enterobacter cloacae* complex isolates in the same faecal screening sample. A third had *bla*_NDM-1_-positive *E*. *coli* and *E*. *cloacae* complex isolates identified in two different specimens (wound secretion and urine, respectively) within a one-month period. The fourth patient yielded two *bla*_NDM-1_-positive *K*. *pneumoniae* strains with unrelated STs from specimens taken 21 months apart.

### Increasing number of CPE identified during the study period from a high proportion of clinical isolates

CPE isolates were identified in 14 of 22 clinical microbiology laboratories representing all health regions in Norway. The number of CPE cases per year, diversity of carbapenemase variants and species increased during the study period (Table 1), but with a trend towards dominance of NDM and OXA-48-like carbapenemase variants and increasing number of carbapenemase-producing *E*. *coli*. Fifty-six percent of the patients were male. The patient age ranged from 3–96 years (mean 63 and median 66 years). The majority of CPE were isolated from urine (*n* = 22, 37%), blood culture (*n* = 9, 15%) and faecal screening (*n* = 9, 15%).

**Table 1:** Time-line and distribution of identified CPEs and carbapenemase variants. No. of isolates in parenthesis.

Year	No. of isolates	No. of cases^a^	*Klebsiella* sp.	*E*. *coli*	Other Enterobacteriaceae
2007	3	3	KPC-2 (1), VIM-1 (2)		
2008	6	6	KPC-2 (6)		
2009	2	2	KPC-2 (2)		
2010	8	7	KPC-2 (2), KPC-3 (1), VIM-27 (2), NDM-1 (1)	NDM-1 (1)	KPC-2 (1)
2011	4	4	KPC-2 (2), NDM-1+OXA-181 (1), OXA-48 (1)		
2012	16	14	KPC-2 (1), VIM-1 (1), NDM-1 (2), NDM-7 (1), OXA-245 (1)	VIM-29 (1), NDM-1 (1), NDM-5 (1), NDM-7 (1), OXA-48 (2)	NDM-1 (3), IMI-9 (1)
2013	8	7	KPC-3 (1), NDM-1 (2), OXA-48 (1), OXA-245 (1)	NDM-1 (1),OXA-48 (2)	
2014	12	10	KPC-2 (2), NDM-1 (2), OXA-48 (1), OXA-162 (1)	VIM-4 (1), NDM-1 (1), IMP-26 (1), OXA-181 (1)	KPC-2 (1), NDM-1 (1)
Total 2007–2014	59	53	KPC-2 (16), KPC-3 (2), VIM-1 (3), VIM-27 (2), NDM-1 (7), NDM-7 (1), NDM-1+OXA-181 (1), OXA-48 (3), OXA-162 (1), OXA-245 (2)	VIM-4 (1), VIM-29 (1), NDM-1 (4), NDM-4 (1), NDM-7 (1), IMP-26 (1), OXA-48 (4), OXA-181 (1)	KPC-2 (2), IMI-9 (1), NDM-1 (4)

^a^ Patients identified with multiple CPE defined as a single case.

### Association with travel or hospitalization abroad

Thirty-three patients (62%) had a known history of travel and/or hospitalization abroad (Table 2). Sixteen patients (30%) reported no travel or hospitalization abroad and for four patients (8%), no information was obtained. With respect to the non-direct import cases, eight cases were associated with secondary spread from imported cases. This included six cases associated with a previously described, small but long-term outbreak of *bla*_KPC-2_-positive *K*. *pneumoniae*/*E*. *cloacae* complex in 2007–2010 [50]. In addition, two other intra-hospital transmissions of *bla*_KPC-2_-positive *K*. *pneumoniae* [28] and *bla*_VIM-27_-positive *K*. *pneumoniae* were observed involving one additional patient in each case.

**Table 2:** Distribution of isolates according to association with importation.

Country	No. of isolates	Species	Sequence type (ST)	Carbapenemase
Greece	7	*K*. *pneumoniae*	ST258	KPC-2
	1	*K*. *pneumoniae*	ST147	VIM-27
India	1	*K*. *pneumoniae*	ST11	NDM-1
	1	*K*. *pneumoniae*	ST17	NDM-1
	1^a^	*K*. *pneumoniae*	ST147	NDM-1
	1	*E*. *coli*	ST101	NDM-7
	1	*E*. *coli*	ST131	NDM-1
	1	*E*. *coli*	ST410	NDM-1
Turkey	1	*K*. *pneumoniae*	ST273	VIM-1
	1	*K*. *variicola*	ST981	OXA-48
	1	*E*. *coli*	ST38	OXA-48
Serbia^b^	1	*K*. *pneumoniae*	ST17	NDM-1
	1	*P*. *stuartii*	-	NDM-1
	1	*P*. *mirabilis*	-	NDM-1
	1	*Citrobacter* sp.	-	NDM-1
Spain	1	*K*. *pneumoniae*	ST11	OXA-245
	1	*K*. *quasipneumoniae*	ST1466	VIM-1
	1	*E*. *cloacae* complex	ST635	IMI-9
Morocco	1	*K*. *pneumoniae*	ST405	OXA-48
	1	*K*. *pneumoniae*	ST11	OXA-245
Thailand	1	*E*. *coli*	ST405	OXA-48
	1	*E*. *coli*	ST6355	VIM-29
Brazil	1	*K*. *pneumoniae*	ST855	KPC-2
United Arab Emirates	1	*K*. *pneumoniae*	ST336	NDM-7
Syria/Jordan	1	*E*. *coli*	ST410	VIM-4
Jamaica	1	*E*. *cloacae* complex	ST456	KPC-2
Pakistan	1	*E*. *coli*	ST617	NDM-1
Romania	1	*K*. *pneumoniae*	ST525	NDM-1+OXA-181
Sri Lanka	1	*K*. *pneumoniae*	ST101	NDM-1
USA	1	*K*. *pneumoniae*	ST258	KPC-3
Unknown	1	*K*. *pneumoniae*	ST187	OXA-48
	2	*E*. *coli*	ST38	OXA-48
	1	*E*. *coli*	ST95	IMP-26
Norway (no reported overseas travel)	9^c^^,^ ^d^	*K*. *pneumoniae*	ST258	KPC-2
	1	*K*. *pneumoniae*	ST14	OXA-162
	1^a^	*K*. *pneumoniae*	ST37	NDM-1
	1^e^	*K*. *pneumoniae*	ST147	VIM-27
	1^c^	*K*. *pneumoniae*	ST461	KPC-2
	1	*K*. *pneumoniae*	ST2134	VIM-1
	1	*E*. *coli*	ST410	OXA-181
	1	*E*. *coli*	ST636	NDM-5
	1^f^	*E*. *coli*	ST681	NDM-1
	1^f^	*E*. *cloacae* complex	ST92	NDM-1
	1^c^	*E*. *cloacae* complex	ST484	KPC-2

^a^ Two *bla*_NDM-1_-positive *K*. *pneumoniae* isolates, one ST147 and one ST37, were isolated from the same patient. The isolates were identified 21 months apart where the first detection was associated with importation, but not for the second detection.

^b^ All four *bla*_NDM-1_-positive isolates were isolated from the same patient.

^c^ Six *K*. *pneumoniae* ST258, one *K*. *pneumoniae* ST461 and one *E*. *cloacae* complex ST484, all *bla*_KPC-2_-positive, were associated with a long-term outbreak [50]. The first case (*K*. *pneumoniae* ST258 with *bla*_KPC-2_) of the outbreak were associated with import from Greece.

^d^ One *bla*_KPC-2_-positive *K*. *pneumoniae* ST258 associated with intra-hospital transmission (first case associated with import from Greece)[28].

^e^ The *bla*_VIM-27_-positive isolate were associated with a case of intra-hospital transmission (first case associated with import from Greece).

^f^ Both isolates identified from the same patient.

### Bacterial species and carbapenemase diversity

Overall *Klebsiella* spp. (*K*. *pneumoniae*, *n* = 36; *Klebsiella variicola n* = 1; *Klebsiella quasipneumoniae n* = 1) were dominant, followed by *E*. *coli* (*n* = 14), *E*. *cloacae* complex (*n* = 4) and single isolates of *P*. *stuartii*, *P*. *mirabilis* and *Citrobacter* sp. (Table 1 and S1 Table). The most dominant carbapenemase gene family was *bla*_KPC_, found in *K*. *pneumoniae* (*n* = 18) and *E*. *cloacae* complex (*n* = 2), followed by *bla*_NDM_ identified in *K*. *pneumoniae* (*n* = 8), *E*. *coli* (*n* = 6), *E*. *cloacae* complex (*n* = 1), *P*. *stuartii* (*n* = 1), *P*. *mirabilis* (*n* = 1) and *Citrobacter* sp. (*n* = 1). *bla*_VIM_ was identified in *K*. *pneumoniae* (*n* = 4), *E*. *coli* (*n* = 2) and *K*. *quasipneumoniae* (*n* = 1) while *bla*_OXA-48-like_ was identified in *K*. *pneumoniae* (*n* = 5), *E*. *coli* (*n* = 5) and *K*. *variicola* (*n* = 1). In addition, we identified one *K*. *pneumoniae* isolate harbouring both *bla*_NDM_ and *bla*_OXA-48-like_ and single isolates with *bla*_IMI_ (*E*. *cloacae* complex) and *bla*_IMP_ (*E*. *coli*). With respect to KPC, KPC-2 (*n* = 18) was the most predominant allele with the closest KPC-3 (*n* = 2) variant detected in only two isolates. The remaining carbapenemase genes encoded three different variants of NDM (NDM-1, *n* = 16; NDM-7, *n* = 2; and NDM-5, *n* = 1), four OXA-48-like (OXA-48, *n* = 7; OXA-181, *n* = 2; OXA-245, *n* = 2 and OXA-162, *n* = 1) and four VIM (VIM-1, *n* = 3; VIM-27, *n* = 2; VIM-4, *n* = 1; and VIM-29, *n* = 1). The single isolates with *bla*_IMI_ and *bla*_IMP_ encoded IMI-9 and IMP-26, respectively.

### Bacterial population structure and linkage to specific carbapenemase alleles

**Fig 1:**
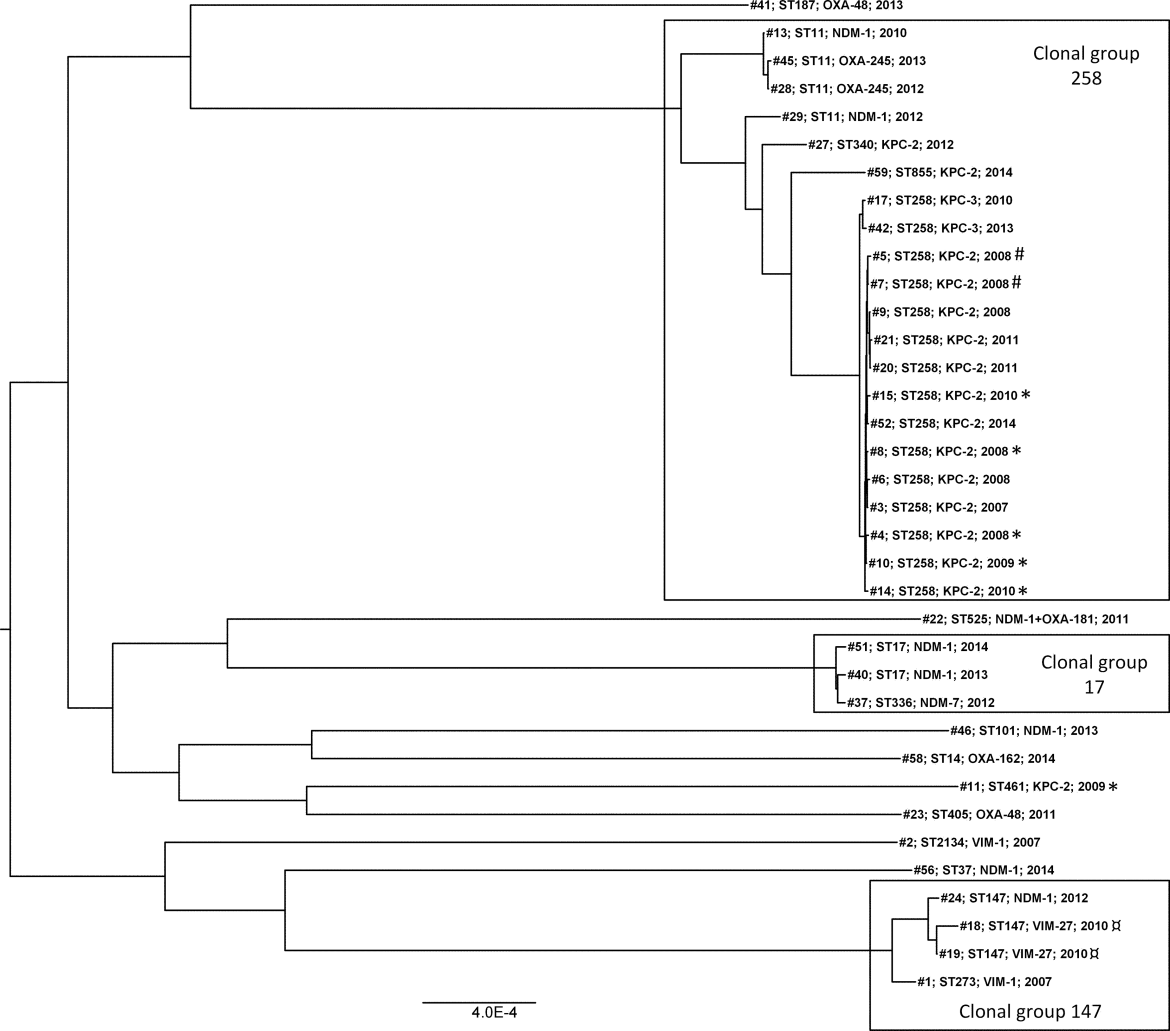
Phylogenetic tree of *K*. *pneumoniae* isolates based on alignment of concatenated sequences of the 694 cgMLST scheme of *K*. *pneumoniae* [22]. The tree was constructed in RAxML [49] and visualized using FigTree (http://tree.bio.ed.ac.uk/software/figtree/). Clonal groups with >1 isolates are boxed. Sequence type (ST), carbapenemase gene and year of isolation is indicated for each isolate. Isolates associated with the long-term outbreak [50] and the two occurrences of intra-hospital transmission are labelled *, # and ¤, respectively.

MLST and cgMLST (Fig 1) showed that *K*. *pneumoniae* was dominated by KPC-producing clonal group (CG) 258, more specifically ST258 (*n* = 15) and its single locus variants (SLV) ST855 (*n* = 1) and ST340 (*n* = 1). The CG258 cluster comprised 21 isolates and included nearly all KPC-producers (*n* = 17) in addition to four ST11 isolates carrying *bla*_NDM-1_ (*n* = 2) or *bla*_OXA-245_ (*n* = 2) genes. Outside CG258, *bla*_KPC_ was only identified in one isolate belonging to ST461. Among the *K*. *pneumoniae* isolates cgMLST identified two other clusters represented by more than one isolate: one representing CG147 and including ST147 with *bla*_VIM-27_ (*n* = 2) or *bla*_NDM-1_ (*n* = 1) and ST273 with *bla*_VIM-1_ (*n* = 1), and one representing CG17 including ST17 with *bla*_NDM-1_ (*n* = 2) and ST336 with *bla*_NDM-7_ (*n* = 1). The remaining *K*. *pneumoniae* isolates represented genetically diverse single strains harbouring *bla*_NDM-1_ (ST37 and ST101), *bla*_NDM-1_ + *bla*_OXA-181_ (ST525), *bla*_OXA-48_ (ST187 and ST405), *bla*_OXA-162_ (ST14) and *bla*_VIM-1_ (ST2134). The *K*. *quasipneumoniae* isolate carrying *bla*_VIM-1_ belonged to ST1466 and the *K*. *variicola* with *bla*_OXA-48_ belonged to ST981.

Ten diverse genetic backgrounds were identified among the *E*. *coli* isolates (*n* = 14). None of the STs were SLVs or double locus variants (DLVs) of any other. Only ST38 (*n* = 3) and ST410 (*n* = 3) were represented by >1 isolate. All three ST38 isolates carried *bla*_OXA-48_, while the three ST410 strains harboured each a different carbapenemase gene (*bla*_NDM-1_, *bla*_VIM-4_ or *bla*_OXA-181_). The remaining strains were genetically diverse and carried various carbapenemase genes/variants: *bla*_NDM-1_ (ST131, ST617 and ST681), *bla*_NDM-5_ (ST636), *bla*_NDM-7_ (ST101), *bla*_OXA-48_ (ST405), *bla*_VIM-29_ (ST6355) and *bla*_IMP-26_ (ST95).

The four carbapenemase-producing *E*. *cloacae* complex isolates were all of different STs: ST456 and ST484 both with *bla*_KPC-2_, ST92 with *bla*_NDM-1_ and ST635 with *bla*_IMI-9_. All STs were defined as singletons (no SLVs) by BURST analysis of the *E*. *cloacae* MLST database (http://pubmlst.org/ecloacae/, last accessed 24.06.2016).

### Antimicrobial susceptibility profile and performance of phenotypic methods for detection of CPE

All isolates were multidrug-resistant (MDR) according to the definitions by Magiorakos *et al*. [51]. (Table 3 and S1 Table). One isolate, a *bla*_NDM-1_-positive *P*. *stuartii* was non-susceptible to all relevant antimicrobial agents tested. Overall fosfomycin and colistin were the most active antimicrobial agents with 85% and 79% of the isolates being susceptible when excluding *P*. *mirabilis* and *P*. *stuartii* isolates which are intrinsically resistant to colistin [52] (Table 3). Seven of the twelve colistin-resistant isolates were *K*. *pneumoniae* ST258 with *bla*_KPC-2_ (*n* = 6) or *bla*_KPC-3_ (*n* = 1). The other colistin-resistant isolates included *K*. *pneumoniae* ST525 with *bla*_NDM-1_ + *bla*_OXA-181_, *K*. *pneumoniae* ST147 with *bla*_NDM-1_, *K*. *pneumoniae* ST336 with *bla*_NDM-7_, *E*. *cloacae* complex ST635 with *bla*_IMI-9_ and *E*. *cloacae* complex ST456 with *bla*_KPC-2_.

**Table 3:** Antimicrobial resistance profiles of CPE isolates according to species and carbapenemase variant.

		Percent non-susceptible (I+R)^a^																
Species	Carbapenemase	TZP	MEC	CXM	CTX	CAZ	ATM	MEM	ETP	IPM	GEN	AMK	TOB	CIP	TGC	SXT	CST	FOS
*Klebsiella* spp.	KPC (*n* = 18)	100	100	100	100	100	100	83	100	50	28	78	83	94	83	72	39	6
	VIM (*n* = 5)	100	100	100	100	100	40	60	100	80	0	40	100	100	40	80	0	0
	NDM (*n* = 9)^b^	100	11	100	100	100	100	89	100	67	78	89	100	89	56	78	33	11
	OXA-48-like (*n* = 6)	100	0	67	50	67	50	17	100	33	17	0	50	83	83	33	0	33
*E*. *coli*	VIM/IMP (*n* = 3)	67	67	100	100	100	100	33	67	67	100	100	100	33	33	100	0	33
	NDM (*n* = 6)	100	17	100	100	100	83	33	100	83	83	83	83	67	0	33	0	0
	OXA-48-like (*n* = 5)	100	0	100	100	100	100	20	100	20	80	0	80	60	20	80	0	0
*E*. *cloacae* complex	KPC (*n* = 2)	100	-^c^	100	100	100	100	100	100	100	50	50	50	100	50	50	50	100
	NDM (*n* = 1)	100	-	100	100	100	100	100	100	100	100	100	100	100	100	100	0	100
	IMI (*n* = 1)	0	-	0	0	0	100	0	100	100	0	0	0	0	100	0	100	0
*P*. *stuartii*	NDM (*n* = 1)	100	-	-	100	100	100	100	100	100	100	100	100	100	-	100	-	100
*P*. *mirabilis*	NDM (*n* = 1)	0	100	100	100	100	100	0	0	100	100	100	100	100	-	100	-	0
*Citrobacter* spp.	NDM (*n* = 1)	100	-	100	100	100	100	0	100	100	100	100	100	100	100	100	0	0
Total^d^		95	53	95	93	95	88	59	97	61	51	63	83	83	58	68	21	15

^a^ according to EUCAST clinical breakpoint table v. 6.0. TZP: piperacillin-tazobactam; MEC: mecillinam; CXM: cefuroxime; CTX: cefotaxime; CAZ: ceftazidime; AZT: aztreonam; MEM: meropenem; ETP: ertapenem; IPM: imipenem; GEN: gentamicin; AMK: amikacin; TOB: tobramycin; CIP: ciprofloxacin; TGC: tigecycline; SXT: trimethoprim-sulfamethoxazole; CST: colistin; FOS: fosfomycin.

^b^ includes one isolate co-harboring *bla*_NDM-1_ and *bla*_OXA-181_.

^C^ “-”indicates lack of clinical breakpoint or intrinsic resistance according to EUCAST Expert Rules on Intrinsic Resistance and Exceptional Phenotypes v.3.1 (http://www.eucast.org/).

^d^ calculations excludes species/antibiotic combinations with intrinsic resistance.

High levels of non-susceptibility were observed to aminoglycosides (gentamicin, 51%; amikacin, 63%; and tobramycin, 83%), tigecycline (58%) and ciprofloxacin (83%).

With respect to the carbapenems, 41% were susceptible to meropenem, 39% to imipenem and 3% to ertapenem. All isolates had meropenem and ertapenem MIC values above the EUCAST screening breakpoint for carbapenemase detection (http://www.eucast.org/fileadmin/src/media/PDFs/EUCAST_files/Resistance_mechanisms/EUCAST_detection_of_resistance_mechanisms_v1.0_20131211.pdf) (S1 Table). For imipenem nine isolates had MIC values below the screening breakpoint. There was no clear correlation between carbapenemase variant and susceptibility to meropenem and imipenem with the exception that among the isolates harbouring *bla*_OXA-48-like_ (excluding the strain with both *bla*_NDM-1_ and *bla*_OXA-181_) 9/11 and 8/11 were susceptible to meropenem and imipenem, respectively. As expected, a high level of resistance was observed against other β-lactams (Table 3 and S1 Table). Three isolates: one *K*. *pneumoniae* (*bla*_OXA-48_), one *K*. *variicola* (*bla*_OXA-48_) and the *bla*_IMI-9_-positive *E*. *cloacae* complex isolate were susceptible to extended-spectrum cephalosporins (cefotaxime, ceftazidime and cefuroxime) and aztreonam. Interestingly, all OXA-48-like-positive *E*. *coli* and *Klebsiella* spp. as well as 83% and 89% of NDM-positive *E*. *coli* and *Klebsiella* spp. isolates, respectively were susceptible to mecillinam. Nine (15%) of the isolates tested negative for carbapenemase-production with the in-house Carba NP test (S1 Table), including six *bla*_NDM-1_-positive isolates (*E*. *coli n* = 2, *P*. *stuartii*, *P*. *mirabilis*, *Citrobacter* sp. and *K*. *pneumoniae*), two *bla*_OXA-48-like_-positive isolates (*E*. *coli* and *K*. *pneumoniae*) and one *E*. *cloacae* complex isolate (*bla*_IMI-9_). The KPC, MBL and OXA-48 confirm kit correctly identified the presence of either an MBL or KPC in all relevant isolates except for one *bla*_NDM-1_-positive *P*. *mirabilis* strain (S1 Table). The single *bla*_IMI-9_-positive *E*. *cloacae* complex isolate also showed significant synergy with boronic acid only. With the exception of the isolate harbouring both *bla*_NDM-1_ and *bla*_OXA-181_, where synergy was observed between meropenem and dipicolinic acid, no synergy was observed with the β-lactamase inhibitors for all *bla*_OXA-48_-like-positive isolates. Moreover, with the exception of two isolates, all *bla*_OXA-48–like_-positive isolates showed no zones of inhibition around the temocillin tablet, which may indicate the presence of OXA-48-like carbapenemases according to the manufacturer’s guidelines. The meropenem-meropenem/EDTA gradient strip correctly identified all MBL-positive isolates, with the exception of the *K*. *pneumoniae* strain positive for both *bla*_NDM-1_ and *bla*_OXA-181_ where the test was inconclusive (S1 Table).

### Association with other antibiotic resistance determinants

*Bla*_CTX-M_ and specifically *bla*_CTX-M-15_ were the most common ESBL variants identified and were mainly associated with *K*. *pneumoniae* and *E*. *coli* isolates with *bla*_NDM_ (10/15 isolates) or *bla*_OXA-48-like_ (8/11 isolates) and *E*. *coli* isolates with *bla*_VIM_ (2/2 isolates) (S1 Table). *Bla*_CTX-M_ were not identified in *bla*_KPC_-positive *K*. *pneumoniae* isolates. One *E*. *coli* isolate with *bla*_OXA-48_ harboured both *bla*_CTX-M-14_ and *bla*_CTX-M-15_. *bla*_CTX-M-15_ was also identified in one *bla*_KPC-2_- and one *bla*_NDM-1_-positive *E*. *cloacae* complex. *Bla*_CMY_ (*n* = 12) were the most common plasmid-mediated AmpC variants identified with *bla*_CMY-6_ particularly associated with *bla*_NDM_ (*n* = 9). The two *bla*_OXA-48-like_-positive *Klebsiella* spp. isolates that were susceptible to extended-spectrum cephalosporins and aztreonam were negative for ESBL and plasmid-mediated AmpC genes.

In addition to various genes encoding aminoglycoside-modifying enzymes, the 16S rRNA methylase genes *rmtC* and *armA*, were identified in eight and five isolates, respectively (S1 Table). With the exception of the single isolate of *E*. *coli* with *bla*_IMP-26_, *armA* and *rmtC* were only associated with isolates harbouring *bla*_NDM-1_. In *Klebsiella* spp. insertional disruption of *mgrB* [53] associated with colistin resistance was identified in seven *K*. *pneumoniae* isolates (S1 Table). Insertional disruption of *mgrB* was also observed in two clinically colistin susceptible (MIC = 1 mg/L) *K*. *pneumoniae* isolates. One *K*. *pneumoniae* isolate with a disrupted *mgrB* also carried a nonsense mutation in *pmrB* leading to a truncated PmrB. Two colistin-resistant *K*. *pneumoniae* isolates had mutations in *pmrA* resulting in amino acid substitutions of G53C and D86E in one, and G53C in the other. In one colistin-resistant *Klebsiella* spp. isolate (MIC >8 mg/L) no previously described colistin resistance determinants were identified. The strain had mutations in *pmrA* (PmrA E57G) and *pmrB* (PmrB T246A) compared with the colistin-susceptible *K*. *pneumoniae* strain MGH 78578 [54], but neither mutation has been linked with colistin resistance and PmrB T246A is commonly found in *K*. *pneumoniae* [54]. No mutations were identified in *phoP*, *phoQ* or the *mgrB* promoter for this isolate. The plasmid-mediated colistin resistance genes *mcr-1* [9], *mcr-2* [10], *mcr-3* [12], *mcr-4* [13] and *mcr-5* [14] were not detected.

All *E*. *coli*, *K*. *pneumoniae* and *E*. *cloacae* complex isolates with high-level ciprofloxacin resistance (MIC ≥32 mg/L) harboured mutations in both *gyrA* and *parC* (S1 Table). In addition, various plasmid-mediated quinolone resistance determinants were identified, including *aac(6’)-Ib-cr* (*n* = 24), *qnrB1* (*n* = 8), *qnrB4* (*n* = 1), *qnrB19* (*n* = 2), *qnrD* (*n* = 1) and *qnrS1* (*n* = 8).

## Discussion

The main objective of this study was to gain a better understanding of the molecular epidemiology associated with the emergence of CPE in Norway. As observed in other Nordic countries [26, 27, 32–36] the emergence of CPE in Norway is also mainly associated with importation, highlighting the importance of targeted screening of patients hospitalized abroad and patients with a recent travel history to a country with a high prevalence of CPE. A relatively low number of cases (15%) were identified through faecal screening in contrast to Sweden (74,5%) and France (59.8%) [26, 55]. This difference is most likely due to dissimilarities in the use of targeted screening and that CPE screening in Norway was not fully implemented in the study period. This could also explain why a higher proportion of CPE cases in Sweden (81%) were associated with import [26]. Revised recommendations for infection prevention and control, including indications for screening for CPE, were introduced in Norway in August 2015 and in the first six months of 2016, 63% of CPE cases were identified through faecal screening. The occurrence of one long-term outbreak and two separate incidences of secondary transmission further highlights the importance of rapid implementation of infection prevention and control measures before confirmation of CPE if patients have risk factors (e.g. hospitalization abroad) or when an MDR isolate is identified.

The diversity of species and genetic backgrounds observed is probably due to the high degree of importation from a variety of countries (Table 2). Several studies have shown that the dissemination of resistance genes among clinical strains of Enterobacteriaceae is often associated with high-risk clones and the linkage between specific genetic backgrounds and resistance genes [2, 21, 56]. The cgMLST analysis of *K*. *pneumoniae* isolates showed that the observed epidemiology reflects the current global epidemiology (Fig 1), where *bla*_KPC-2/-3_ spread is primarily driven by strains associated with CG258 (and more specifically, ST258). In contrast, ST11 (a member of CG258, and a single locus variant of ST258) has been shown to be associated with a diversity of carbapenemase genes including *bla*_KPC_, *bla*_NDM_, *bla*_VIM_ and *bla*_OXA-48-like_ in different geographical regions [2, 57, 58]. Accordingly, the four ST11 strains in this study harboured either *bla*_NDM-1_ (*n* = 2) or *bla*_OXA-245_ (*n* = 2). Notably, cgMLST has shown that ST11 and ST340 represent a genetic sublineage within CG258 [22]. Isolates with *bla*_NDM_ and *bla*_VIM_ belonging to two other globally dispersed high-risk CGs like CG17 and CG147 [2] were also identified. The identification of *bla*_VIM-1_ and *bla*_OXA-48_ in *K*. *quasipneumoniae* and *K*. *variicola*, respectively shows that these *Klebsiella* species also contribute to the dissemination of carbapenemase genes and infections as both isolates were associated with infection. *K*. *variicola* have been shown to be frequently associated with bloodstream infections and associated with higher mortality than *K*. *pneumoniae* [59].

All three *E*. *coli* ST38 isolates harboured *bla*_OXA-48_, which is consistent with previous observations showing a prevalent linkage of ST38 to *bla*_OXA-48_ in a large collection of clinical isolates from European and North-African countries [23]. In contrast, the three *E*. *coli* isolates belonging to ST410 were associated with different carbapenemase genes (*bla*_NDM-1_, *bla*_VIM-4_ or *bla*_OXA-181_) indicating the ability of this genetic background to maintain different plasmids and resistance genes. ST410 *E*. *coli* isolates have also previously been identified harbouring *bla*_KPC-2_ [60]. The global dissemination of *bla*_NDM_ has so far not been linked to specific high-risk clones or epidemic plasmids [21] and this is also reflected among the five *bla*_NDM_-positive *E*. *coli* isolates, which belonged to five different genetic backgrounds. However, one strain belonged to the international high-risk clone ST131 [21] and another to ST101, which has previously been found to be associated with *bla*_NDM_ and other carbapenemases in several countries (e.g. Bangladesh [61], USA [62], Canada [63, 64] and Bulgaria [65]).

CPE frequently exhibit MDR or XDR phenotypes, limiting treatment options [1, 4]. This was also observed in our strain collection (Table 2 and S1 Table) due to the association with a wide variety of other acquired resistance genes, including 16S rRNA methylase genes conferring high-level broad-spectrum aminoglycoside resistance [66] and chromosomal mutations/insertions resulting in ciprofloxacin and colistin resistance (S1 Table). The mechanism(s) behind colistin resistance in one *K*. *pneumoniae* strain and the colistin-resistant *E*. *cloacae* isolates remains to be determined. Interestingly, a high prevalence of susceptibility to mecillinam among OXA-48- and NDM-producing *E*. *coli* and *K*. *pneumoniae* isolates was observed. Marrs *et al*. also showed high levels of *in vitro* susceptibility to mecillinam among NDM-producing *E*. *coli* and *K*. *pneumoniae* isolates from Pakistan [67], suggesting that mecillinam could have a role in the treatment of uncomplicated urinary tract infections caused by OXA-48- or NDM-producing *E*. *coli* or *K*. *pneumoniae* [68].

Rapid identification of CPE is essential for timely implementation of enhanced infection control measures to reduce transmission of CPE and prevent infections [3]. As observed in previous studies [69, 70] false-negative results (15%) for carbapenemase production were observed with the in-house version of the Carba NP test, particularly with NDM- and OXA-48-like-producing isolates. Identification of OXA-48-like-producers can be particularly challenging due to their relatively low level of activity against carbapenems and the lack of specific inhibitors [71]. The relatively high number of false-negative Carba NP results could also be due to the media used. In our study, colonies for the Carba NP test were harvested from MH agar and Literacka et al have recently reported that MH agar from different companies were associated with false-negative results for MBL-producers [72]. High-level resistance to temocillin is a sensitive and specific indicator for the presence of OXA-48-like enzymes [73]. All *bla*_OXA-48-like_–positive isolates in our collection showed high-level resistance (MIC>128 mg/L) to temocillin, but several isolates harboring *bla*_VIM_ and *bla*_NDM_ also had temocillin MIC >128mg/L showing that testing for synergy with metal chelators (e.g. EDTA or dipicolinic acid) is necessary to discriminate between isolates with OXA-48 and MBLs.

## Conclusions

The low prevalence of clinical CPE in Norway is consistent with the general low level of antimicrobial resistance compared with other countries. The relatively low level of antibiotic consumption and the use of narrow spectrum antibiotics [74] have probably contributed to this situation. The low prevalence is also reflected in the epidemiology of Norwegian CPE; mainly associated with importation, exhibiting a broad diversity of genetic backgrounds and carbapenemase variants that mirror the global epidemiology. Only a few cases of secondary spread also support this notion. In order to limit the infection pressure brought by increasing travel and globalization, continued emphasis must be put on diagnostic capabilities, surveillance and infection control.

## Supporting information

S1 Table Strain collection and associated phenotypic and molecular data. (XLSX)
